# Impact of gastric emptying to the glycemic and insulinemic responses to a 75‐g oral glucose load in older subjects with normal and impaired glucose tolerance

**DOI:** 10.14814/phy2.12204

**Published:** 2014-11-20

**Authors:** Laurence G. Trahair, Michael Horowitz, Chinmay S. Marathe, Kylie Lange, Scott Standfield, Christopher K. Rayner, Karen L. Jones

**Affiliations:** 1 Discipline of Medicine, The University of Adelaide, Adelaide, South Australia, Australia; 2 NHMRC Centre of Research Excellence in Translating Nutritional Science to Good Health, The University of Adelaide, Adelaide, South Australia, Australia

**Keywords:** Gastric emptying, glucose, IGT, insulin, insulin sensitivity, OGTT, older subjects

## Abstract

The majority of studies relating to the oral glucose tolerance test (OGTT) have not taken gastric emptying (GE), which exhibits a substantial inter‐individual variation, into account. We sought to evaluate the impact of GE, on the glycemic and insulinemic responses to a 75‐g oral glucose load in older subjects with normal and impaired glucose tolerance. Eighty‐seven healthy ‘older’ subjects (47F, 40M; age 71.0 ± 0.5 year) were given a drink comprising of 75‐g glucose and 150 mg C^13^‐acetate made up to 300 mL with water on a single occasion. Exhaled breath was obtained for analysis of ^13^CO_2_ and calculation of the 50% GE time (T_50_). Blood glucose, serum insulin and plasma glucagon‐like peptide‐1 (GLP‐1) and glucose‐dependent insulinotropic peptide (GIP) were measured, and the insulin sensitivity index (ISI), and the disposition index (DI), were calculated. Thirty‐one subjects had normal glucose tolerance (NGT) and 46 had impaired glucose tolerance (IGT). Blood glucose at *t* = 60 min and *t* = 120 min were related inversely to ISI (*P* < 0.001) and DI *P* < 0.001). The rise in blood glucose at *t* = 60 min was related inversely to the T_50_ in all subjects (*P* < 0.01), and those with IGT (*P* < 0.001), but not NGT. There were no significant relationships between the blood glucose at *t* = 120 min with the T_50_, but in both groups the change in blood glucose from baseline at *t* = 180 min was related (NGT:* P* < 0.001; IGT:* P* < 0.001) to the T_50_. We conclude that in NGT and IGT, the effect of GE on both the ‘early’ and ‘late’ glycemic responses to a 75‐g oral glucose load is complementary to that of insulin sensitivity.

## Abstract (precis)

The 75‐g oral glucose tolerance test is regarded as the ‘gold standard’ for the diagnosis of impaired glucose tolerance and diabetes, however, it is subject to substantial variability. This is likely to be accounted for not only by differences in insulin resistance and beta cell function but also by gastric emptying, which exhibits a wide inter‐individual variation. Our study characterized the impact of gastric emptying on glycemic, insulinemic, and incretin responses to a 75‐g oral glucose load in subjects with normal glucose tolerance and impaired glucose tolerance. We demonstrated that both the rate of gastric emptying and insulin sensitivity are independent, and complementary, determinants of both the ‘early’ and ‘late’ responses to an oral glucose tolerance test in healthy older subjects.

## Introduction

The World Health Organisation 75‐g oral glucose tolerance test (OGTT) is regarded as the ‘gold standard’ for the diagnosis of impaired glucose tolerance (IGT) and diabetes (American Diabetes A 2014) and is also predictive of the development of type 2 diabetes (Abdul‐Ghani et al. 2010). The OGTT does, however, exhibit substantial variability (Mooy et al. 1996) and there are uncertainties about the diagnostic value of the traditional 120‐min glucose, as opposed to the 60‐min value (Abdul‐Ghani et al. 2008, 2009). In particular, the 60‐min plasma glucose may correlate better with insulin secretion and resistance (Abdul‐Ghani et al. 2008, 2009).

The variability in the OGTT is likely to be accounted for, in part, by gastric emptying (GE) which, in health, exhibits a wide interindividual variation (Collins et al. 1983) so that nutrients, including glucose, usually enter the small intestine at an overall rate of 1–4 kcal/min, primarily as a result of inhibitory feedback arising from the small intestine (Brener et al. 1983). This interindividual variation is increased in longstanding diabetes because of the high prevalence of delayed (Horowitz et al. 2001), and occasionally more rapid, GE (Phillips et al. 1992). Studies in small cohorts have established that GE is a major determinant of the initial (from ~15 to 60 min) glycemic response to oral glucose and carbohydrate‐containing meals in healthy volunteers (Horowitz et al. 1993, 1996; Corvilain et al. 1995; Schwartz et al. 1995; O'Donovan et al. 2005), type 2 patients with both normal and disordered GE (Jones et al. 1995; Stevens et al. 2011) and hypertensive patients (Phillips et al. 1997). It has also been suggested that more rapid GE may predispose to the development of type 2 diabetes (Phillips et al. 1992). The dependence of postprandial glycemia on GE provides a rationale for the use of dietary and/or pharmacological (most recently, ‘short‐acting’ glucagon‐like peptide‐1 (GLP‐1) agonists) interventions which slow GE to reduce postprandial glycemic excursions (Linnebjerg et al. 2008).

In contrast to the above, there is much less information about the relationship of the blood glucose level at 120 min during an OGTT with GE (Horowitz et al. 1993; Corvilain et al. 1995). Horowitz et al. reported that, in health, this relationship is positive, rather than inverse, presumably reflecting the insulin levels achieved earlier (Horowitz et al. 1993). This also appears to apply to the blood glucose at 180 min (Corvilain et al. 1995). To our knowledge, there is no information about the impact of GE on the 120‐min blood glucose in patients with IGT, or type 2 diabetes. Furthermore, studies which have evaluated the effect of GE on the glycemic response to glucose have not assessed insulin secretion, or sensitivity. The incretin hormones, GLP‐1 and glucose‐dependent insulinotropic peptide (GIP), modulate the glycemic response to oral carbohydrate (Nauck et al. 1986) and their secretion may be influenced by the rate of GE (Pilichiewicz et al. 2007).

We hypothesized that GE would have a complementary effect to that of insulin sensitivity on the glycemic and insulinemic responses to a 75‐g oral glucose load. This hypothesis could potentially be addressed by manipulating GE in isolation, which is problematic. Given the substantial interindividual variation in GE, we have quantified GE as well as both ‘early’ and ‘late’ glycemic responses, and insulin sensitivity in a cohort of older subjects with either normal glucose tolerance (NGT) or IGT.

## Materials and Methods

### Subjects

Eighty‐seven healthy ‘older’ subjects (47 female and 40 male, mean age 71.0 ± 0.5 years [range: 65–90 years], body mass index [BMI] 26.0 ± 0.3 kg/m^2^ [range: 20.3–30.5 kg/m^2^]), were recruited by advertisements placed in the local hospital campus and newspaper. Prior to their inclusion, demographic information and a basic medical history were obtained. Subjects with a history of gastrointestinal disease or surgery, known diabetes, significant respiratory or cardiac disease, alcohol abuse (consumption >20 g/day) or epilepsy, were excluded. Any medication was withheld for 24 h prior to the study.

### Protocol

In each subject, concurrent measurements of GE, blood glucose, serum insulin, and plasma GLP‐1 and GIP were obtained on a single study day, which commenced at 0830 h after an overnight fast from solids for 14 h and liquids for 12 h. Upon arrival, an intravenous (IV) cannula was inserted into an antecubital vein for blood sampling while the subject was supine. The subject was then seated and allowed to ‘rest’ for 15–30 min before consuming a drink containing 75‐g glucose and 150‐mg C^13^‐acetate (Cambridge Isotope Laboratories, Tewksbury, MA), made up to 300 mL with water, within 3 min; *t* = 0 min was defined as the time of completion of the drink. Exhaled breath samples were collected in hermetically sealed 10‐mL tubes (Exetainer, Buckinghamshire, England) prior to the ingestion of the drink (*t* = −3 min), every 5 min for the first hour, and then every 15 min for the subsequent 3 h, for assessment of GE. Venous blood samples, for measurement of blood glucose, serum insulin, plasma GLP‐1, and plasma GIP, were obtained in tubes containing EDTA at *t* = −3, 15, 30, 45, 60, 90, 120, 180, and 240 min, centrifuged at 1490 *g* for 15 min and plasma, or serum, separated, and stored at −70°C. At *t* = 240 min the IV cannula was removed and the subject offered a light lunch prior to them leaving the laboratory. The protocol was approved by the Research Ethics Committee of the Royal Adelaide Hospital, and each subject provided written, informed consent. All experiments were carried out in accordance with the Declaration of Helsinki.

### Gastric emptying

The ^13^CO_2_ concentration in breath samples was measured by an isotope ratio mass spectrometer (ABCA 20/20; Europa Scientific, Crewe, UK) with an online gas chromatographic purification system. The gastric 50% emptying time (T_50_) was calculated (Ghoos et al. 1993).

### Blood glucose, serum insulin, plasma GLP‐1, and plasma GIP

Blood glucose (mmol/L) was determined using a portable glucometer (Medisense Companion 2 meter, Medisense Inc., Waltham, MA) and each subject classified, according to WHO (Alberti and Zimmet 1998) criteria, as having NGT (fasting blood glucose <6.1 mmol/L, and 2 h <7.8 mmol/L), impaired fasting glucose (IFG) (fasting blood glucose <7.0 mmol/L, but >6.1 mmol/L), IGT (2 h blood glucose <11.1 mmol/L, but >7.8 mmol/L), or diabetes (fasting blood glucose ≥7.0 mmol/L and/or 2 h blood glucose ≥11.1 mmol/L) (Alberti and Zimmet 1998).

Serum insulin was measured by ELISA immunoassay (10‐1113, Mercodia, Uppsala, Sweden). The sensitivity was 1.0 mU/L and the coefficient of variation (CV) was 2.6% within, and 7.6% between, assays (Trahair et al. 2012). Total GLP‐1 was measured by radioimmunoassay (GLPIT‐36HK, Millipore, Billerica, MA). Minimum detectable limit was 3 pmol/L, intra and interassay CVs were 7.7% and 9.4%, respectively (Trahair et al. 2012). Plasma GIP was measured by radioimmunoassay. Minimum detectable limit was 2 pmol/L, interassay CV was 8.7%, and intraassay CV was 5.0% (Wishart et al. 1992).

### Insulin sensitivity and disposition index

The insulin sensitivity index (ISI) of Matsuda and DeFronzo (1999) was calculated as follows: 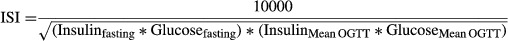


where insulin is in mU/L and glucose is in mg/dL. The ratio of the incremental changes from baseline in insulin and glucose at 30 min (∆I30/∆G30) was calculated as a measure of *β*‐cell function (Phillips et al. 1994). Insulin secretion, corrected for *β*‐cell function (the oral disposition index [DI]), was calculated as the product of the Matsuda index and *β*‐cell function (∆I30/∆G30*ISI) (Kim et al. 2012).

### Statistical analysis

For blood glucose, serum insulin, and plasma GLP‐1 and GIP, changes from baseline and total areas under the curve (AUC) at *t* = 60, 120, 180, and 240 min were calculated. Changes in each variable over time as were evaluated with a one‐way repeated measures ANOVA. Data in subjects with NGT and IGT were compared (excluding subjects with IFG alone, or diabetes) using Student's t‐test. Pearson's correlation was used to evaluate relationships between variables. A multiple regression model was used to assess the determinants of the blood glucose at *t* = 60, 120, and 180 min. In this model, covariates included the T_50_, ISI, and DI. Results of the multiple regressions are reported as adjusted *R*^2^ (*R*^2^_Adj_). Semipartial correlations are reported for the variables within each regression (*R*_Part_). A *P* < 0.05 was considered significant in all analyses. The statistical analysis was supervised and reviewed by a professional biostatistician (KL). Data are presented as mean values ± SEM.

## Results

The studies were well tolerated and there were no adverse events. Thirty‐one subjects had NGT, 32 had IGT, and 14 had both IFG and IGT; that is, 46 had IGT. Eight had IFG alone and two had diabetes; these 10 subjects were excluded from the analysis, resulting in a cohort of 77 subjects. Demographic variables in the subjects are provided in Table 1. There were no differences in age or BMI between the groups with IGT and NGT. In three subjects (one NGT, two IGT), GE data were unavailable due to degradation of the breath samples; in one the *t* = 180 and 240‐min blood samples and in another the *t* = 240‐min sample, were unavailable as the cannula was not patent. In two subjects, the insulin sample at *t* = 240 min was lost.

**Table 1.** Demographic variables in the whole cohort (*n* = 77), subjects with NGT (*n* = 31) and IGT (*n* = 46)

	Whole group	NGT	IGT
Age (years)	70.8 ± 0.5	69.8 ± 0.7	71.5 ± 0.7
Sex	39F, 38M	12F, 19M	27F, 19M
BMI (kg/m^2^)	26.0 ± 0.3	25.6 ± 0.5	26.2 ± 0.4
GE T_50_ (min)	140.5 ± 4.3 (*n* = 74)	141.8 ± 6.5 (*n* = 30)	139.5 ± 5.7 (*n* = 44)

Data are mean ± SEM. BMI, body mass index; GE, gastric 50% emptying time (T_50_); IGT, impaired glucose tolerance; NGT, normal glucose tolerance.

### Gastric emptying

The GE T_50_ was 140.5 ± 4.3 min (*n* = 74, range: 95–256 min). There was no difference in the T_50_ between the groups with IGT and NGT (139.5 ± 5.7 min vs. 141.8 ± 6.5 min, *P* = 0.80).

### Blood glucose, serum insulin, plasma GLP‐1, and plasma GIP

**Figure 1.**
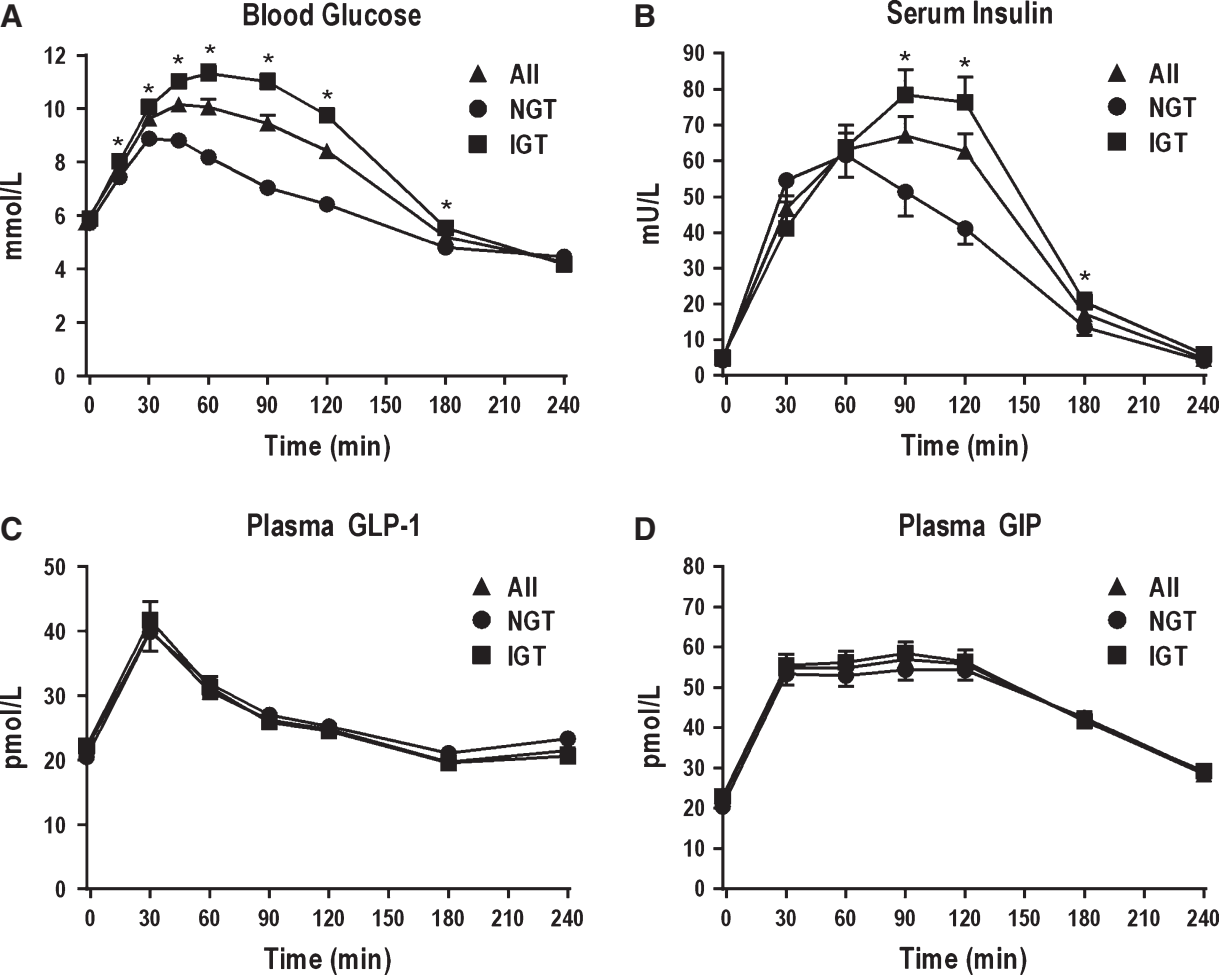
Blood glucose (A), serum insulin (B), plasma GLP‐1 (C), and plasma GIP (D) immediately before and after a 75‐g oral glucose load in all subjects (▲, *n* = 77), subjects with normal glucose tolerance (●, *n* = 31) and those with impaired glucose tolerance (± impaired fasting glucose) (■, *n* = 46) (*P < 0.05 NGT vs. IGT).

Blood glucose increased following the drink (*P* < 0.001) and was less than baseline at both *t* = 180 and 240 min (*P* < 0.001 for both) (Fig. 1A). In the group with IGT when compared with those with NGT, blood glucose was greater at baseline, *t* = 15, 30, 45, 60, 90, 120, and 180 min (*P* < 0.05 for all), but not at *t* = 240 min (*P* = 0.34), and the AUC for blood glucose was greater at *t* = 60, 120, 180, and 240 min (*P* < 0.001 for all) (Fig. 1A).

Serum insulin increased following the drink (*P* < 0.001) and had returned to baseline by *t* = 240 min (Fig. 1B). In the group with IGT when compared to those with NGT, serum insulin was greater at *t* = 90, 120, and 180 min (*P* < 0.05 for all) (Fig. 1B).

There was an increase in plasma GLP‐1 following the drink (*P* < 0.001), with a peak at ~*t* = 30 min and levels returning to baseline by *t* = 180 min (Fig. 1C). There was a sustained increase in plasma GIP (*P* < 0.001) until *t* = 120 min (Fig. 1D). There were no differences in absolute levels, or the AUC, for plasma GLP‐1 or GIP between the groups with NGT or IGT (Fig. 1D).

### Insulin Sensitivity Index, *β*‐cell function, and Disposition Index

In NGT, ISI (8.6 ± 0.9 vs. 6.0 ± 0.5, *P* < 0.01), ∆I30/∆G30 (15.2 ± 1.8 vs. 9.4 ± 0.9, *P* < 0.05), and DI (110.1 ± 13.1 vs. 54.3 ± 9.2, *P* < 0.001) were greater when compared with those with IGT.

### Relationships between blood glucose, serum insulin, insulin sensitivity, and incretin hormones

#### Glucose at 60 min

In the whole group (*n* = 77) the blood glucose at *t* = 60 min was related directly to the fasting blood glucose (*R* = 0.50, *P* < 0.001) and insulin (*R* = 0.28, *P* < 0.005), as well as the rise in insulin between *t* = 0–60 min (*R* = 0.33, *P* < 0.005) and inversely to the ISI (*R* = −0.48, *P* < 0.001) and DI (*R* = −0.68, *P* < 0.001). Similarly, the rise in blood glucose at *t* = 60 min was related inversely to the ISI (*R* = −0.45, *P* < 0.001) and DI (*R* = −0.68, *P* < 0.001).

In subjects with NGT (*n* = 31) the blood glucose at *t* = 60 min was related to fasting glucose (*R* = 0.38, *P* < 0.05), fasting insulin (*R* = 0.36, *P* < 0.05), and the rise in insulin between *t* = 0–60 min (*R* = 0.40, *P* < 0.05). In IGT (*n* = 46) the blood glucose at *t* = 60 min was also related to fasting glucose (*R* = 0.35, *P* < 0.05) and the rise in insulin between *t* = 0–60 min (*R* = 0.40, *P* < 0.01). In both groups, the blood glucose at *t* = 60 min was related inversely to both the ISI (NGT: *R* = −0.36, *P* < 0.05; IGT: *R* = −0.45, *P* < 0.005) and DI (NGT: *R* = −0.66, *P* < 0.001; IGT: *R* = −0.60, *P* < 0.001).

#### Glucose at 120 min

In the whole group, the blood glucose at *t* = 120 min was related to the fasting blood glucose (*R* = 0.45, *P* < 0.001), insulin (*R* = 0.29, *P* < 0.01), and insulin at *t* = 120 min (*R* = 0.43, *P* < 0.001) and inversely to the ISI (*R* = −0.43, *P* < 0.001) and DI (*R* = −0.53, *P* < 0.001). Similarly, the change in blood glucose at *t* = 120 was related inversely to both the ISI (*R* = −0.37, *P* < 0.005) and DI (*R* = −0.50, *P* < 0.001).

In NGT, the blood glucose at *t* = 120 min was not related to fasting serum insulin, but there was a relationship with serum insulin at *t* = 120 min (*R* = 0.38, *P* < 0.05); in IGT, blood glucose at *t* = 120 min was related to fasting serum insulin (*R* = 0.33, *P* < 0.05), but not the serum insulin at *t* = 120 min. In contrast, the blood glucose at 120 min was related inversely to both the ISI (NGT: *R* = −0.45, *P* < 0.05; IGT: *R* = −0.31, *P* < 0.05) and DI (NGT: *R* = −0.58, *P* < 0.001; IGT: *R* = −0.35, *P* < 0.05). In NGT, there was no relationship between the change in blood glucose at *t* = 120 min and the ISI (*R* = −0.20, *P* = 0.17), however, there was an inverse relationship with DI (*R* = −0.31, *P* < 0.05) and in IGT, the change in blood glucose at *t* = 120 min was related inversely to both the ISI (*R* = −0.38, *P* < 0.05) and DI (*R* = −0.53, *P* < 0.005).

#### GLP‐1 and GIP

There were no significant relationships between either the absolute, or rises in plasma GLP‐1 or GIP with blood glucose at *t* = 60 or 120 min in the whole group, or in NGT or IGT. In the whole group, there was a relationship between plasma GLP‐1 at *t* = 60 min and insulin at *t* = 60 min (*R* = 0.35, *P* < 0.005), which was significant in IGT (*R* = 0.44, *P* < 0.005), but not NGT (*R* = 0.20, *P* = 0.29). There was no relationship between plasma GIP and insulin at *t* = 60 min.

### Relationships with gastric emptying

#### Glucose and insulin at 60 min

**Figure 2.**
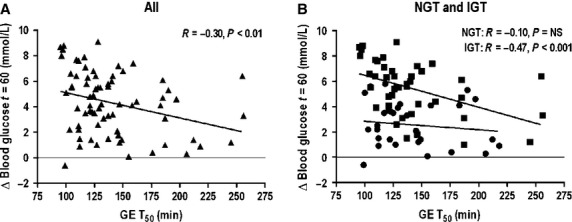
Relationships between the rise in blood glucose between *t* = 0–60 min and the T_50_ in (A) all subjects (*n* = 74, *R* = −0.30, *P* < 0.01), (B) NGT (●, *n* = 30, *R* = 0.10, *P* = NS) and IGT (■, *n* = 44, *R* = −0.47, *P* < 0.001).

In the whole group (*n* = 74), there were inverse relationships between rises in both blood glucose (*R* = −0.30, *P* < 0.01, Fig. 2A) and serum insulin (*R* = −0.23, *P* < 0.05) between *t* = 0–60 min and the T_50_. Similarly, there were inverse relationships between the absolute blood glucose (*R* = −0.27, *P* < 0.05) and serum insulin (*R* = −0.23, *P* < 0.05) at *t* = 60 min and the T_50_.

In the group with NGT (*n* = 30) neither the rises in, or absolute, blood glucose (*R* = −0.10, *P* = 0.54, Fig. 2B) and serum insulin (*R* = −0.27, *P* = 0.14) between *t* =0–60 min were related to the T_50_. In contrast, in IGT (*n* = 44) both the rise in blood glucose (*R* = −0.47, *P* < 0.001, Fig. 2C), absolute blood glucose (*R* = −0.43, *P* < 0.005) and the AUC (*R* = −0.36, *P* < 0.05) from *t* = 0–60 min, but not serum insulin were related inversely to the T_50._

#### Glucose and insulin at 120 min

**Figure 3.**
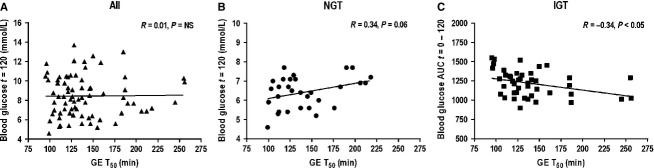
Relationships between absolute blood glucose at *t* = 120 min and the T_50_ in (A) all subjects (*n* = 74, *R* = 0.01, *P* = NS) and (B) NGT (*n* = 30, *R* = 0.34, *P* = 0.06) and between blood glucose AUC 0–120 min and the T_50_ in (C) IGT (*n* = 44, *R* = −0.34, *P* < 0.05).

In the whole group, there was no relationship between the absolute, change in, or AUC for, blood glucose or serum insulin at *t* = 120 min and the T_50_ (Fig. 3A), however, in subjects with NGT there was a trend for a relationship between both the change in blood glucose between *t* = 0–120 min (*R* = 0.34, *P* = 0.06) and the absolute blood glucose at *t* = 120 min (*R* = 0.34, *P* = 0.06, Fig. 3B), but not the AUC for blood glucose at *t* = 120 min (*R* = 0.03, *P* = 0.87) and the T_50_. In the group with IGT, the AUC for blood glucose (but not the absolute or change in blood glucose) at *t* = 120 min was related inversely to the T_50_ (*R* = −0.34, *P* < 0.05, Fig. 3C).

#### Glucose and insulin at 180 min

**Figure 4.**
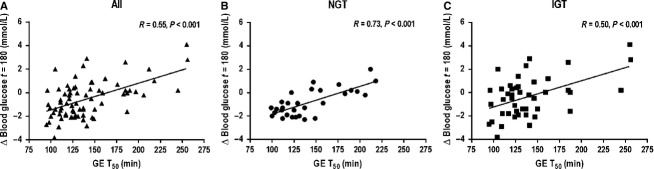
Relationships between the change from baseline for blood glucose between *t* = 0–180 min and the T_50_ in (A) all subjects (*n* = 74, *R* = 0.55, *P* < 0.001), (B) NGT group (*n* = 30, *R* = 0.73, *P* < 0.001), and (C) the IGT group (*n* = 44, *R* = 0.50, *P* < 0.001).

In contrast to the rise, in the whole group there was a relationship between the change in blood glucose at *t* = 180 min (*R* = 0.55, *P* < 0.001, Fig. 4A) and the T_50_. The absolute blood glucose at *t* = 180 min was also related to the T_50_ (*R* = 0.56, *P* < 0.001). Similarly, in the whole group both the change in (*R* = 0.54, *P* < 0.001), and absolute (*R* = 0.43, *P* < 0.001) serum insulin at *t* = 180 min were related to the T_50_.

In the NGT group, the absolute blood glucose (*R* = 0.74, *P* < 0.001), and serum insulin (*R* = 0.48, *P* < 0.01) at *t* = 180 min, and change in blood glucose (*R* = 0.73, *P* < 0.001, Fig. 4B) and serum insulin (*R* = 0.55, *P* < 0.005) between *t* = 0–180 min, were related to the T_50_. In the IGT group, the absolute blood glucose (*R* = 0.53, *P* < 0.001) and serum insulin (IGT: *R* = 0.44, *P* < 0.001) at *t* = 180 min and the change in blood glucose (*R* = 0.50, *P* < 0.001, Fig. 4C) and serum insulin (*R* = 0.57, *P* < 0.001) between *t* = 0–180 min, were related to the T_50_.

### Determinants of the absolute and rises in blood glucose

#### Glucose at 60 min

In the whole group (*n* = 74), a multivariable model incorporating the ISI, DI, and T_50_, with the absolute blood glucose at *t* = 60 min as the dependent variable, was significant (*P* < 0.001), with individual significance for the ISI (*P* < 0.05), DI (*P* < 0.01) and the T_50_ (*P* < 0.05) (Table 2). In the group with NGT (*n* = 30) an identical model was significant (*P* < 0.001), however, the DI was the only significant variable in this model (*P* < 0.001). In the group with IGT (*n* = 44), this model was significant (*P* < 0.001) with significance for the T_50_ (*P* < 0.05), DI (*P* < 0.005), and a trend for the ISI (*P* = 0.08).

**Table 2.** Relationships of glycemia with gastric emptying (T_50_), insulin sensitivity index (ISI) and disposition index (DI)

Blood glucose time	Variable	All subjects (*n* = 74)	NGT (*n* = 30)	IGT (*n* = 44)
60 min	Overall model	*R*^2^_Adj_ = 0.52, *P* < 0.001	*R*^2^_Adj_ = 0.38, *P* < 0.001	*R*^2^_Adj_ = 0.45, *P* < 0.001
	T_50_	*R*_Part_ = −0.21, *P* < 0.05	*R*_Part_ = −0.20, *P* = 0.18	*R*_Par_ = −0.29, *P* < 0.05
	ISI	*R*_Part_ = −0.19, *P* < 0.05	*R*_Part_ = −0.14, *P* = 0.35	*R*_Par_ = −0.21, *P* = 0.08
	DI	*R*_Part_ = −0.49, *P* < 0.01	*R*_Part_ = −0.57, *P* < 0.001	*R*_Par_ = −0.35, *P* < 0.005
CFB 60 min	Overall model	*R*^2^_Adj_ = 0.52, *P* < 0.001	*R*^2^_Adj_ = 0.38, *P* < 0.005	*R*^2^_Adj_ = 0.44, *P* < 0.001
	T_50_	*R*_Part_ = −0.24, *P* < 0.05	*R*_Part_ = −0.22, *P* = 0.15	*R*_Par_ = −0.33, *P* < 0.01
	ISI	*R*_Part_ = −0.16, *P* = 0.06	*R*_Part_ = −0.12, *P* = 0.44	*R*_Par_ = −0.14, *P* = 0.22
	DI	*R*_Part_ = −0.51, *P* < 0.01	*R*_Part_ = −0.57, *P* < 0.001	*R*_Par_ = −0.37, *P* < 0.005
120 min	Overall model	*R*^2^_Adj_ = 0.32, *P* < 0.001	*R*^2^_Adj_ = 0.41, *P* < 0.001	*R*^2^_Adj_ = 0.12, *P* = 0.06
	T_50_	*R*_Part_ = 0.07, *P* = 0.48	*R*_Part_ = 0.27, *P* = 0.07	*R*_Par_ = 0.07, *P* = 0.64
	ISI	*R*_Part_ = −0.22, *P* < 0.05	*R*_Part_ = −0.28, *P* = 0.06	*R*_Par_ = −0.20, *P* = 0.17
	DI	*R*_Part_ = −0.38, *P* < 0.01	*R*_Part_ = −0.42, *P* < 0.01	*R*_Par_ = −0.23, *P* = 0.11
CFB 120 min	Overall model	*R*^2^_Adj_ = 0.27, *P* < 0.001	*R*^2^_Adj_ = 0.34, *P* < 0.005	*R*^2^_Adj_ = 0.04, *P* = 0.19
	T_50_	*R*_Part_ = 0.06, *P* = 0.56	*R*_Part_ = 0.27, *P* = 0.08	*R*_Par_ = 0.04, *P* = 0.80
	ISI	*R*_Part_ = −0.18, *P* = 0.08	*R*_Part_ = −0.23, *P* = 0.14	*R*_Par_ = −0.10, *P* = 0.52
	DI	*R*_Part_ = −0.38, *P* < 0.001	*R*_Part_ = −0.40, *P* < 0.01	*R*_Par_ = −0.23, *P* = 0.13
180 min	Overall model	*R*^2^_Adj_ = 0.35 *P* < 0.001	*R*^2^_Adj_ = 0.53, *P* < 0.001	*R*^2^_Adj_ = 0.31, *P* < 0.001
	T_50_	*R*_Part_ = 0.58, *P* < 0.001	*R*_Part_ = 0.76, *P* < 0.001	*R*_Part_ = 0.58, *P* < 0.001
	ISI	*R*_Part_ = −0.09, *P* = 0.37	*R*_Part_ = −0.12, *P* = 0.34	*R*_Part_ = 0.01, *P* = 0.97
	DI	*R*_Part_ = −0.16, *P* < = 0.09	*R*_Part_ = 0.16, *P* = 0.22	*R*_Part_ = −0.25, *P* = 0.06
CFB 180 min	Overall model	*R*^2^_Adj_ = 0.29, *P* < 0.001	*R*^2^_Adj_ = 0.52, *P* < 0.001	*R*^2^_Adj_ = 0.26, *P* < 0.005
	T_50_	*R*_Part_ = 0.56, *P* < 0.001	*R*_Part_ = 0.75, *P* < 0.001	*R*_Part_ = 0.54, *P* < 0.001
	ISI	*R*_Part_ = −0.01, *P* = 0.96	*R*_Part_ = −0.08, *P* = 0.54	*R*_Part_ = 0.10, *P* = 0.47
	DI	*R*_Part_ = −0.13, *P* = 0.21	*R*_Part_ = 0.19, *P* = 0.19	*R*_Part_ = −0.24, *P* = 0.08

CFB, change from baseline; DI, disposition index; IGT, impaired glucose tolerance; ISI, insulin sensitivity index; NGT, normal glucose tolerance; *R*^2^_Adj_, adjusted *R*^2^; *R*_Part_, semipartial correlation; T_50_, gastric 50% emptying time.

In the whole group, a model incorporating the ISI, DI, and T_50_, with the rise in blood glucose at *t* = 60 min as the dependent variable, was significant (*P* < 0.001), with significance for the DI (*P* < 0.01) and T_50_ (*P* < 0.05), and a trend for the ISI (*P* = 0.06). In the group with NGT, this model was significant (*P* < 0.005), with the DI (*P* < 0.001) as the only significant variable. In the group with IGT this model was significant (*P* < 0.001), with significance for the DI (*P* < 0.005) and T_50_ (*P* < 0.01) only (Table 2).

#### Glucose at 120 min

In the whole group, a multivariable model incorporating the ISI, DI, and T_50_, with the absolute level of blood glucose at *t* = 120 min as the dependent variable, was significant (*P* < 0.001), with significance for the ISI (*P* < 0.05) and DI (*P* < 0.01), but not the T_50_. In the group with NGT this model was significant (*P* < 0.001) with significance for the DI (*P* < 0.01), and a trend for ISI (*P* = 0.06) and T_50_ (*P* = 0.07). In the group with IGT, there was a trend for this model to be significant (*P* = 0.06).

In the whole group, a model incorporating the ISI, DI, and T_50_, with the change in blood glucose at *t* = 120 min as the dependent variable, was significant (*P* < 0.001), with the DI (*P* < 0.001) as the only significant variable. In the group with NGT this model was significant (*P* < 0.005) with significance for the DI (*P* < 0.01) and a trend for the T_50_ (*P* = 0.08). In the group with IGT, this model was not significant (Table 2).

#### Glucose at 180 min

In the whole group, a multivariable model incorporating the ISI, DI, and T_50_, with the absolute level of blood glucose at *t* = 180 min as the dependent variable, was significant (*P* < 0.001), with significance for the T_50_ only (*P* < 0.001). In the group with NGT this model was significant (*P* < 0.001) with the T_50_ as the only significant variable (*P* < 0.001). In the group with IGT this model was significant (*P* < 0.001), with significance for the T_50_ (*P* < 0.001), and a trend for the DI (*P* = 0.06).

In the whole group, a model incorporating the ISI, DI, and T_50_, with the change in blood glucose at *t* = 180 min as the dependent variable, was significant (*P* < 0.001), with significance for the T_50_ only (*P* < 0.001). In the group with NGT this model was significant (*P* < 0.001) with significance for the T_50_ only (*P* < 0.001). In the group with IGT this model was significant (*P* < 0.005) with a significance for the T_50_ (*P* < 0.001), and a trend for the DI (*P* = 0.08) (Table 2).

## Discussion

We have observed that the magnitude of the rise in blood glucose at 60 min is more closely related to GE in subjects with IGT than in those with NGT (i.e., when GE is relatively more rapid, the rise in glucose is proportionally greater), whereas the glycemic response at 120 min tended to be related positively to GE (i.e., T_50_) in subjects with NGT, but was inversely related in those with IGT, probably reflecting the earlier insulinemic response. We have also confirmed that the blood glucose levels at 60 and 120 min following an OGTT are related to insulin sensitivity in healthy older subjects.

Assessment of the relationship of GE with glycemia is complicated by their interdependency. Acute elevations in glycemia slow GE (Fraser et al. 1990), whereas GE is a determinant of glycemia (Horowitz et al. 1993). It has been assumed that the initial rise in blood glucose is modulated primarily by first‐phase insulin secretion and hepatic insulin sensitivity (Reaven et al. 1993). Given its potential diagnostic relevance (Abdul‐Ghani et al. 2008, 2009), we selected the blood glucose value at 60 min to reflect the ‘early’ glycemic response. Our study demonstrates that the relationship of glycemia and GE is time‐dependent, likely reflecting changes in insulin sensitivity and secretion, whereas the blood glucose at 60 min was only significantly related to the rate of GE in the group with IGT. It should be appreciated that in NGT, the smaller variance in blood glucose at 60 min, as well as the earlier peak, may have contributed to the absence of a correlation. Interestingly, at 180‐min, blood glucose levels were related to the rate of GE in both groups and in this case the relationship was inverse, that is, when GE was relatively more rapid, the blood glucose at 180 min was less, presumably reflecting the proportionally greater insulin responses that occurred at earlier time points. Studies, utilizing intraduodenal infusions of glucose, have provided evidence that the relationships between small intestinal glucose delivery and initial glycemic and insulinemic responses are nonlinear in both health (Pilichiewicz et al. 2007) and type 2 diabetes (Ma et al. 2012). That such a relationship was not observed in this study may reflect a lack of subjects with GE rates close to the upper limit of the normal range. With a breath test, the GE T_50_ should be regarded as notional, rather than precise, despite the demonstrated close correlation with scintigraphy (Ghoos et al. 1993), however, it is likely that in the majority of subjects GE was <2 kcal/min, which is associated with only modest glycemic and GLP‐1 responses (Pilichiewicz et al. 2007).

There were no differences in either the GIP, or GLP‐1 responses between the NGT and the IGT groups, consistent with previous observations (Nauck et al. 2004). However, in NGT the maximum glycemic response only modestly exceeded the threshold (8–10 mmol/L) for insulinotropic effects of GIP and GLP‐1 (Toft‐Nielsen et al. 2001), unlike the case in subjects with IGT. Hence, the secretion of GIP and GLP‐1 may be of greater relevance as a compensatory mechanism in the latter group and contribute to hyperinsulinemia. We did not measure plasma glucagon, which is also modulated by both GIP and GLP‐1 in a glucose‐dependant manner (Vilsboll et al. 2003). Insulin sensitivity and glucose disposition are recognized major determinants of the glycemic response to oral glucose (Reaven et al. 1993). We calculated the ISI as described by Matsuda, and the DI adjusted for *β*‐cell function and these were shown to be determinants of the rises in blood glucose at both 60 and 120 min, as would be predicted. It is well established that in cases of NGT, insulin sensitivity may be comparable to that in type 2 patients; however, it is only once the *β*‐cell loses its capacity to compensate for the impaired insulin action that blood glucose concentrations increase.

Our observations are consistent with the concept that GE is a major determinant of the initial glycemic response to carbohydrate‐containing meals in type 2 diabetes and impacts on the overall glycemic response (Jones et al. 1995). It is intuitively likely that GE will assume increased importance in type 2 patients as *β*‐cell function declines; GLP‐1 is of particular relevance given the diminished insulinotropic effects of GIP (Nauck et al. 1993) and studies in type 2 patients employing mixed meals are indicated. While it should be recognized that blood glucose was quantified by glucometer, with its inherent limitations, our study supports the concept that the plasma glucose at 60 min during an OGTT provides clinically meaningful information (Bardini et al. 2010); a cut‐off of 8.6 mmol/L may represent a risk factor for type 2 diabetes (Abdul‐Ghani et al. 2008, 2009; Bardini et al. 2010). However, it may also represent a marker of relatively rapid GE per se. It should also be recognized that our cohort was exclusively >65 years old and that aging is characterized by diminished glucose tolerance, reflecting impairments in insulin sensitivity and *β*‐cell function (Korosi et al. 2001; Chang et al. 2006). There is also a modest slowing of gastric emptying with age, but the rate of emptying usually falls within the normal range for the healthy young (Moore et al. 1983; Horowitz et al. 1984).

We conclude that the rate of GE and insulin sensitivity appear to be independent, and complementary, determinants of both the ‘early’ and ‘late’ responses to an OGTT in healthy older subjects.

## Acknowledgments

The authors would like to acknowledge Max Bellon from the Department of Nuclear Medicine, PET and Bone Densitometry at the Royal Adelaide Hospital for his assistance with the C^13^‐breath test analysis. This study was supported by the National Health and Medical Research Council (NHMRC) of Australia. LGT is supported by an Australian Postgraduate Award, and a Dawes scholarship from the Royal Adelaide Hospital. KLJ's salary is funded by an NHMRC Senior Career Development Award.

## Conflict of Interest

MH has participated in the advisory boards and/or symposia for Novo Nordisk, Sanofi, Novartis, Eli Lilly, Merck Sharp & Dohme, Boehringer Ingelheim, and AstraZeneca/BMS and has received honoraria for this activity. CKR has received research funding from Merck, Eli Lilly and Novartis. None of the other authors has any personal or financial conflict of interest to declare.
